# Biocompatibility and feasibility of VisiPlate, a novel ultrathin, multichannel glaucoma drainage device

**DOI:** 10.1007/s10856-021-06613-8

**Published:** 2021-11-24

**Authors:** Brandon W. Kao, Elana Meer, Thomas A. Barbolt, Richard A. Lewis, Iqbal Ike Ahmed, Vivian Lee, Samuel M. Nicaise, Georgia Griggs, Eydie G. Miller-Ellis

**Affiliations:** 1 Avisi Technologies, Philadelphia, PA USA; 2 University of California San Francisco, San Francisco, CA USA; 3 University of Pennsylvania, Philadelphia, PA USA; 4 Sacramento Eye Consultants, San Francisco, CA USA; 5 Ophthalmology & Vision Sciences, University of Toronto, Toronto, ON Canada

## Abstract

**Background:**

Glaucoma is the leading cause of blindness worldwide. Glaucoma drainage devices and minimally invasive glaucoma surgeries (MIGS) often present with tradeoffs in safety and durability of efficacy. Using a rabbit model, we examined the biocompatibility and feasibility of VisiPlate, a novel, ultrathin, tubeless subconjunctival shunt comprised of a network of microchannels.

**Methods:**

Six naive female New Zealand White rabbits received implants (three only in the right eye with contralateral eye untreated and three in both eyes) composed of a 400-nm-thick aluminum oxide core coated with 2 µm of parylene-C, manufactured with microelectromechanical systems (MEMS) techniques. Tonometry, slit lamp exam, clinical exam, fluorescein patency testing, and histopathology were performed.

**Results:**

VisiPlate demonstrated IOP-lowering of 20–40% compared to baseline at each time point over the course of 3 months in the nine implanted eyes. All eyes developed blebs over the implant, and fluorescein testing demonstrated fluid patency at 22 days post-implantation. Slit lamp and clinical observations showed that VisiPlate was well tolerated, with low levels of conjunctival congestion, conjunctival swelling, aqueous flare, hyphema, and iris involvement from surgery that resolved over time. At sacrifice time points of 93 days and 180 days, the only notable observations were mild levels of conjunctival congestion in implanted eyes. Histopathology showed minimal tissue response and no obvious inflammation, fibrosis, or necrosis around the implant.

**Conclusions:**

The results of this in vivo study demonstrate the biocompatibility and IOP-lowering effect of a multichannel, ultrathin subconjunctival shunt in a rabbit model. The data suggest that VisiPlate may safely enhance aqueous outflow and significantly reduce intraocular pressure.

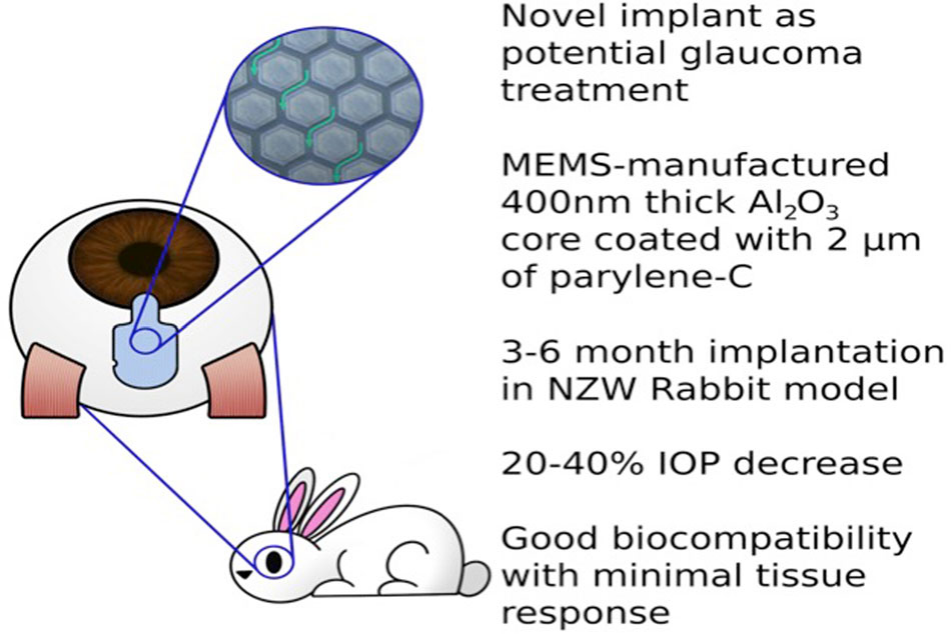

## Introduction

Glaucoma is a group of eye conditions that damage the optic nerve. It is a leading cause of irreversible blindness for people over the age of 60 [1] and affects more than 65 million people globally with projections to increase to 111.8 million by 2040 [2]. The most common form of glaucoma is primary open-angle glaucoma, in which aqueous humor cannot drain properly through the blocked trabecular meshwork. As the aqueous humor accumulates in the eye, it increases intraocular pressure (IOP) and damages the optic nerve, leading to slow, asymptomatic vision loss [3]. Importantly, IOP remains the only modifiable risk factor for glaucoma [4].

Permanent glaucoma drainage devices have been developed to target different severities of disease: tube shunts mainly treat refractory or severe glaucoma, while minimally invasive glaucoma surgeries (MIGS), angle-based procedures that do not result in subconjunctival filtration, are approved for mild-to-moderate glaucoma. Tube shunts are more effective over a 5-year period, and are increasingly used as a first-line filtration surgery, compared to the gold-standard incisional filtration surgery, trabeculectomy- [5]; however, tube shunts have a thick profile, remain invasive, and often reserved as a last line of defense. Meanwhile, subconjunctival microstents (e.g., Xen gel stent, Preserflo) have poor drainage-sustaining capability when compared to tube shunts, often requiring revision procedures like needling [6]. Furthermore, both tube shunts and subconjunctival microstents rely on a single-lumen tube to drain aqueous humor to lower IOP. Single-lumen tubes are prone to occlusion with scar tissue and erosion through healthy tissue, which may necessitate revision surgery [5–9].

A wide range of materials have been used in glaucoma drainage implants. Traditionally, polymer materials such as silicone have been used (Molteno Device, Ahmed Glaucoma Valve, Baerveldt Glaucoma Implant). More recently, materials such as titanium (iStent) [10], poly(styrene-b-isobutylene-b-styrene) (Preserflo) [11], nitinol (Hydrus) [12], polyimide (Cypass) [13], gold (Solx) [14], and parylene-C [15] have provided various degrees of success in reducing tissue reaction and sustainably reducing IOP. While aluminum oxide and parylene-C have been used extensively in medical devices because of their biocompatibility profile [16, 17], there has not been any intraocular use of these materials.

In this study, we present a prototype of a novel, ultrathin, highly flexible, subconjunctival shunt, VisiPlate. The studied VisiPlate is comprised of an 400-nm-thick freestanding aluminum oxide (alumina) corrugated plate coated with a 2-µm-thick layer of parylene-C, making it up to 30× thinner than existing MIGS and 300× thinner than tube shunts. The hexagonal, corrugated structure is based on a plate metamaterial structure developed at the University of Pennsylvania [18] that confers both flexural stiffness and shape recovery properties. The combination of alumina with parylene-C results in a composite material with the resilience to withstand surgical manipulation and physiological forces within the eye, as well as the flexibility and thin profile for optimal tissue biointegration. Altogether, VisiPlate’s material composition and structure facilitates safe, comfortable, and efficacious implants.

VisiPlate is the first glaucoma drainage device to lower IOP through a network of open interconnected microchannels, rather than a single-lumen tube. Microchannel dimensions were designed according to a modified Hagen–Poiseuille equation for rectangular channels to provide adequate flow rates for IOP lowering without hypotony. Aqueous humor in the anterior chamber of the eye flows through VisiPlate to a pocket known as a “bleb” between the conjunctival and scleral tissues in the eye. From there, surrounding tissue gradually reabsorbs the excess fluid, lowering pressure within the eye and protecting the optic nerve. VisiPlate’s redundant channels for pressure release prevent single-end clogging, while its thin profile hinders tissue erosion and the surface area of the plate maintains a drainage space over time.

The purpose of this study is to provide evidence of biocompatibility and efficacy of this device in reducing IOP in in vivo rabbit models.

## Methods

Study protocols involving animals were approved by the IACUC committee of the contract research organizations. During the study, the care and use of animals were conducted in accordance with the regulations of the USDA Animal Welfare Act and in compliance with Absorption Systems California’s (ASC, San Diego, CA) Animal Welfare Assurance (A4282-01) filed with the National Institutes of Health. All surgery and animal housing was performed at the contract research organization Absorption Systems.

### Device

**Fig. 1:**
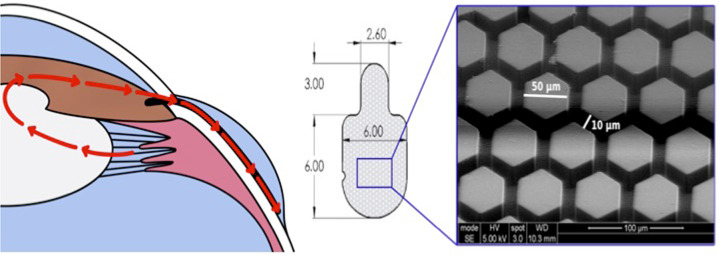
Placement and structure of VisiPlate. **A** Anatomic placement of VisiPlate into the subconjunctival space. **B** Structure and dimensions of VisiPlate in millimeters, with scanning electron micrograph of VisiPlate’s surface topography of hexagonal corrugation and networked channels

VisiPlate devices were constructed using microelectromechanical systems manufacturing techniques on silicon wafers. Photolithography with a patterned chromium mask on glass creates the hexagonal pattern in a layer of photoresist. Dry reactive ion etching etches the pattern into the silicon, creating a mold of hexagonal ribs. Next, atomic layer deposition conformally deposits alumina onto the silicon mold. The silicon is then etched from the alumina. Lastly, individual devices are cut out via laser micromachining and coated with parylene-C using conformal chemical vapor deposition. For this study, device dimensions were scaled down to fit in the smaller rabbit eye (Fig. 1).

### Animals and examination procedures

Only healthy animals without signs of any significant ocular irritation were selected. Animals were acclimated for a period of 40–61 days prior to the study. They were maintained with a 12-h light–dark cycle with free access to food and water. Throughout the in vivo phase of the study, animals were observed daily for any abnormal clinical events. Body weights were recorded at baseline, Week 3, and prior to termination.

Six naive female New Zealand White rabbits were utilized for this study. Three Group 1 animals received device implants, VisiPlate v3.4, in the right eye (OD) while the contralateral left eye (OS) remained untreated. Three Group 2 animals received devices into both eyes, with mitomycin C (MMC) being used during the right eye (OD) implantation procedure.

#### Clinical ophthalmic examinations and slit lamp photographs

Clinical ophthalmic examinations and slit lamp biomicroscopy were performed on both eyes (OU) of all study animals at baseline (prior to device administration), on day 0 immediately after device implantation, and on days 1, 3, 5/7, 14/15, 22, 28 35, 49/51, 62/65, 77, and 89/90. Additional examinations were performed for Group 2 animals on days 12, 16, 124, 149, and 180. These time points were in line with the literature and standard of care for similar in vivo studies [19–21]. General health observations were performed daily.

#### IOP measurement procedure

Animals were acclimated once daily to the IOP measurement procedure for 5 days prior to initiation of the study to habituate the animals to the procedure and to determine baseline IOP levels. IOP measurements were performed with a TonoVet rebound tonometer, at the same time in the early morning (±1 h) by the same technician with at least three measurements taken per eye per each measurement at baseline (prior to device administration), on day 0 immediately after device implantation, and on days 1, 3, 6/7, 10/12, 14/15, 16, 22, 28, 35, 49/51, 62/63, 77, and 90. Additional examinations were performed for Group 2 animals on days 124, 149, and 180.

#### Fluorescein testing procedure

Fluorescein testing to evaluate the passage of aqueous humor from the anterior chamber to the subconjunctival space was performed in all device-implanted eyes (OD in Group 1, OU in Group 2) on day 22. Animals were anesthetized as described above. The anterior chamber was entered with a small (30–31 gauge) needle, and aqueous humor was allowed to drain from the anterior chamber to avoid excessive IOP with continuous IOP monitoring. A second small needle was introduced into the anterior chamber and ~0.5 mL of a 0.01% sodium fluorescein solution in Balanced Saline Solution (BSS) was slowly infused into the anterior chamber over 20 min.

### Implantation procedure

Animals were anesthetized with intramuscular (IM) injections of ketamine hydrochloride (25–51 mg/kg) and xylazine (5–10 mg/kg). Glycopyrrolate (0.01–0.02 mg/kg, IM) was administered concurrently. Atipamezole hydrochloride (1 mg/kg, IM) was used as a reversal agent. After the area was surgically prepped and prior to the surgical procedure, one to two drops of topical proparacaine hydrochloride anesthetic (0.5%) were applied to the animals’ eyes prior to device implantation.

For Group 1 (OD) and 2 (OD), 100 μL of 0.2 mg/mL MMC was injected into the subconjunctival space prior to first incision, exposed for 10 min and then flushed out with BSS. No MMC was used for the left eyes (OS) of Group 2 or any of the eyes in Group 1. A 60 to 90-degree fornix-based conjunctival peritomy was made in the superior-temporal quadrant, with the initial conjunctival incision made 2 mm posterior to the limbus. The length of the subconjunctival pocket was 8 mm from the initial incision. A stab incision into the anterior chamber was made 1 mm from the limbus with a keratome blade to create a scleral tunnel between the subconjunctival pocket and the anterior chamber. The device was grasped using forceps and inserted gently into the subconjunctival pocket. The neck of the implant was guided into the scleral tunnel and into the anterior chamber. The body of the implant was smoothed out to ensure it was properly positioned and lying flat. The implant was anchored to the sclera using 10-0 nylon sutures by passing the suture through the device at each of the corners and the tail. The conjunctiva was closed with 10-0 nylon sutures.

Animals were recovered immediately after device administration. One injection of buprenorphine (0.05 mg/kg SC) was given perioperatively for analgesia. One drop of 0.3% ofloxacin and one drop of 1% prednisolone acetate was applied on day 0 after completion the implantation procedure and then 4× daily on days 1 through 7 after device administration.

### Termination and histological processes

Animals were euthanized via pentobarbital overdose (150 mg/kg, IV) on day 93 (Group 1) or day 180 (Group 2) after the final evaluation. The euthanasia procedure was performed in compliance with the American Veterinary Medical Association Guidelines on Euthanasia.

Tissue collection immediately followed euthanasia. Both eyes (whole globes) were collected from all animals in all groups. Two sutures of different colors were placed to identify the device location. Eyes (with no slits or cuts) were placed into vials containing modified Davidson’s fixative and stored at room temperature for 48 h, then switched to 70% ethanol and shipped to Histion (Everett, WA), a company contracted for processing and histopathological services. Previously embedded paraffin specimens were reverse processed to remove the paraffin, then processed and embedded in MMA, sectioned, cover slipped, and submitted for microscopic analysis to assess inflammation and tissue response to implant (fibrosis and necrosis). Digital images were taken of each slide using a Glissando Slide Scanner coupled with a 20× objective.

In order to allow sufficient striation of the findings to determine possible differences between groups, eyes were categorized according to histomorphologic gradings of: minimal (a histomorphologic change that is just noticeable or a change so minor, small, or infrequent as to warrant no more than the least assignable grade), mild (a histomorphologic change that is a notable, but not a prominent, feature of the tissue), moderate (a histomorphologic change that is a prominent feature of the tissue), and marked (a histomorphologic change that is a predominant feature of the tissue).

## Results

No signs of animals suffering distress were observed throughout both parts of the study, suggesting the device was well tolerated by the rabbits. Device implantation did not affect body weights or general, non-ocular health. Overall, VisiPlate demonstrated both IOP-lowering and biocompatibility over the course of 3 and 6 months with IOP decreasing 20–40% across all non-MMC eyes, and 40–60% across all Group 2 eyes, from baseline and maintained over the 3–6 month periods.

### Clinical observations and slit lamp exam

#### Group 1

By day 1, all treated eyes had developed typical post-implant findings of conjunctival congestion, swelling, iritis, and some degree of hyphema, with particularly large hyphema observed in the right eye (OD) of one animal secondary to iris injury and moderate hemorrhage due to focal laceration by keratome during implantation. Hyphema resolved in some eyes by day 7 and in all eyes after day 14. Aqueous flare resolved after day 3, aside from a single, faint, transient incident on day 22. Pupillary reflex impairment, iris hyperemia, and corneal opacity all resolved after day 3, although a single instance of transient mild iris hyperemia was observed on day 22. Conjunctival congestion, swelling, and/or discharge also improved after day 3, but mild conjunctival congestion and occasional swelling persisted over the implant site only in all three treated eyes. Untreated eyes remained generally free of ocular anomalies, aside from a few instances of mild conjunctival congestion and/or swelling on days 1–3 (Supplementary Table 1).

#### Group 2

By day 1, all Group 2 animals exhibited mild-to-severe conjunctival congestion, swelling, and/or discharge and faint to intense aqueous flare in both eyes (OU) after device implantation (with and without MMC). Most eyes also exhibited mild-to-moderate iris hyperemia, and a subset exhibited impaired pupillary reflex, resolving over the course of the study with intermittent recurrence in two eyes. Conjunctival congestion and swelling persisted throughout the study, though at later time points congestion and swelling were generally limited to the implant site. Aqueous flare and/or fibrin in the anterior chamber recurred throughout the study, appearing frequently in some eyes and occasionally in all eyes, sometimes accompanied by cells in the aqueous humor. Pupillary reflex impairment resolved by day 3, but recurred in several eyes on day 22. Iris adhesions developed starting on day 28, and were associated with iris thinning. However, according to a modified McDonald grading system, iris involvement was still considered to be minimal. **(**Supplementary Table 1) Hyphema was noted in two eyes resolving spontaneously, and three eyes exhibited subconjunctival hemorrhage over the implant (Supplementary Table 2). All of the ocular complications described above were observed to comparable degrees in MMC-treated eyes (OD) and eyes not treated with MMC (OS) with no significant difference in iris involvement. All complications described in Supplementary Table 2.

**Fig. 2:**
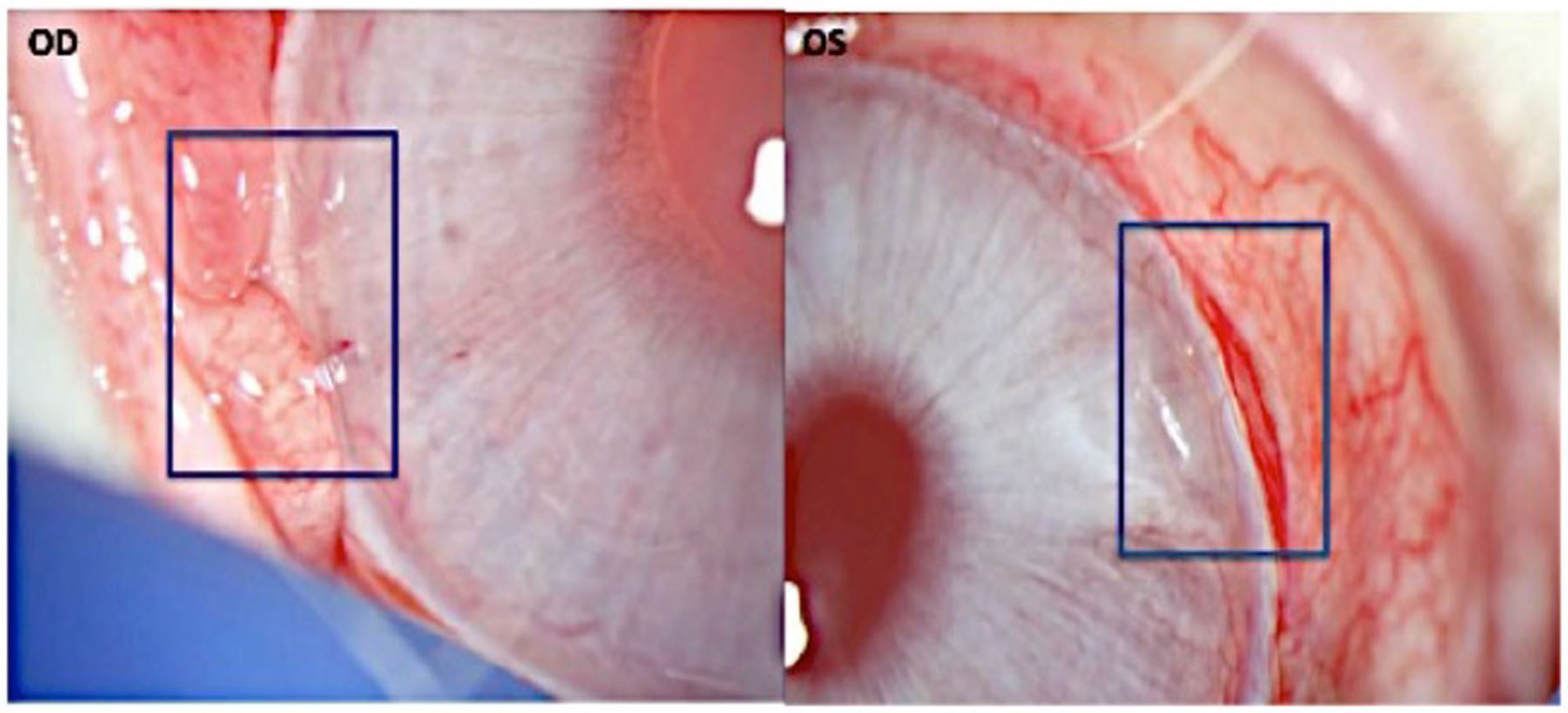
Slit lamp exam photos from day 180 from Group 2 with implanted VisiPlate in the right eye (OD) treated with MMC and the left eye (OS) without MMC. Minimal inflammation was visible with appropriate placement of VisiPlate maintained in the subconjunctival space

All eyes developed blebs over the device implant and on post-implant procedure on day 0. Group 2 eyes treated with MMC exhibited enlarged blebs starting from day 1 after device administration. Blebs appeared white, reflecting vasoconstriction and subsequent ischemia or devascularization of the blebs with revascularization of the bleb around day 35. Bleb size also gradually reduced over time for a few eyes. By day 180, there were no significant differences in bleb appearance or VisiPlate placement between eyes treated with and without MMC on slit lamp exam (Fig. 2).

### IOP measurements

#### Group 1

Right eyes (OD) and left eyes (OS) of Group 1 animals had comparable IOP levels during acclimation. A sharp drop in IOP on day 0 was likely due to the effects of anesthesia and/or the device implantation procedure on that day. Mean IOP values after device implantation in both implanted eyes (OD) and untreated eyes (OS) were decreased below baseline levels (approximately −3 mmHg, or ~20% decrease from baseline). While mean IOP values in untreated eyes (OS) tended to be numerically slightly higher than those in device-implanted eyes (OD), the difference was not significant.

#### Group 2

**Fig. 3:**
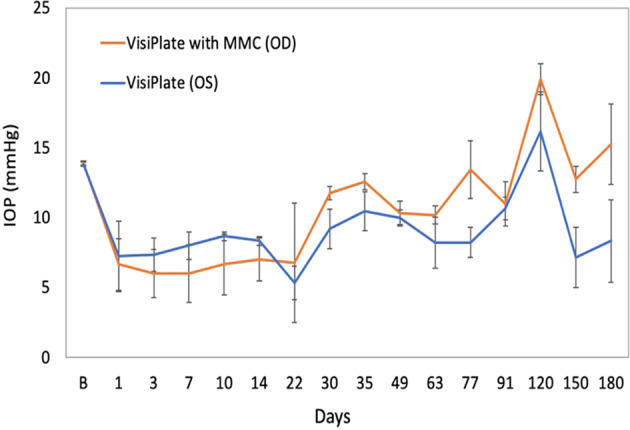
Intraocular pressure measurements showing VisiPlate-implanted eyes with and without MMC injection in Group 2 (*n* = 3), presented as mean ± SE. After device implantation, IOP dropped to around 50% of baseline IOP post-operatively (around −6 to −7 mmHg, or 40–50% decrease from baseline) and remained low for the non-MMC-treated groups. MMC-treated eyes initially exhibited slightly greater IOP reductions (up to −8 mmHg or nearly 60% decrease from baseline) compared to eyes not treated with MMC; however, by month 6, MMC-treated eyes had returned to baseline. There was no significant difference between the MMC and non-MMC groups at any time point. IOPs were significantly lower (paired one-tailed Student’s *t* test, *p* < 0.05) than baseline at days 1, 3, 7, 10, 13, 30, 49, and 63 for the MMC + VisiPlate group, and at days 3, 7, 10, 14, 22, 30, 49, 63, 77, 91, 150 for the VisiPlate only group

Group 2 animals likewise exhibited comparable IOP levels OD and OS during acclimation. After device implantation, a more pronounced decrease in mean IOP levels was observed (around −6 to −7 mmHg, or 40–50% decrease from baseline). MMC-treated eyes initially exhibited slightly greater IOP reductions (up to -8 mmHg, or nearly 60% decrease from baseline) compared to eyes not treated with MMC. However, from Month 1 onward, IOP levels in MMC-treated eyes gradually increased, and by Month 6, IOP values in these eyes had returned to baseline levels. In contrast, significant IOP decreases in eyes not treated with MMC persisted through Month 6 (Fig. 3).

**Fig. 4:**
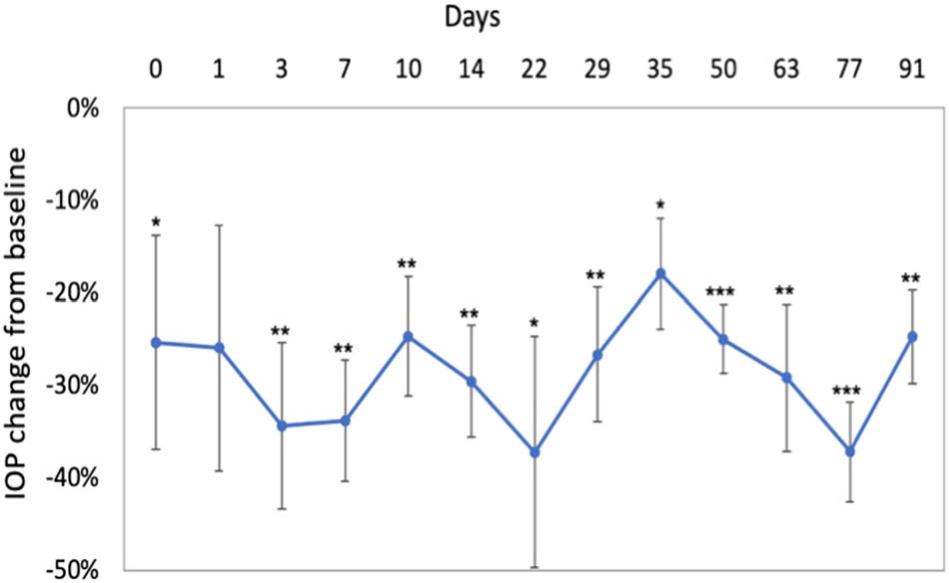
Intraocular pressure decrease from baseline of eyes implanted with VisiPlate alone across all groups (*n* = 6), presented as mean ± SE. There was a significant reduction from baseline rabbit IOP of 15 mmHg exceeding the FDA’s efficacy threshold of 20% IOP reduction from baseline. *p < 0.05, **p < 0.01, ***p < 0.001, paired one-tailed Student’s *t* test

Overall, IOP measurements showing all six eyes implanted with VisiPlate without MMC at 3 months demonstrated a significant reduction, at some points close to 40% reduction, from a baseline IOP of 15 mmHg (Fig. 4).

### Fluorescein testing

Fluorescein testing demonstrated the passage of aqueous humor from the anterior chamber to the subconjunctival space in all examined eyes over 60 min post fluorescein injection. A large, diffuse filtration zone is maintained beyond VisiPlate surface area and beyond dissection zone. There appeared to be no differences in the fluorescein test findings between MMC-treated eyes and eyes not treated with MMC on digital photography and slit lamp.

### Histopathological findings

**Fig. 5:**
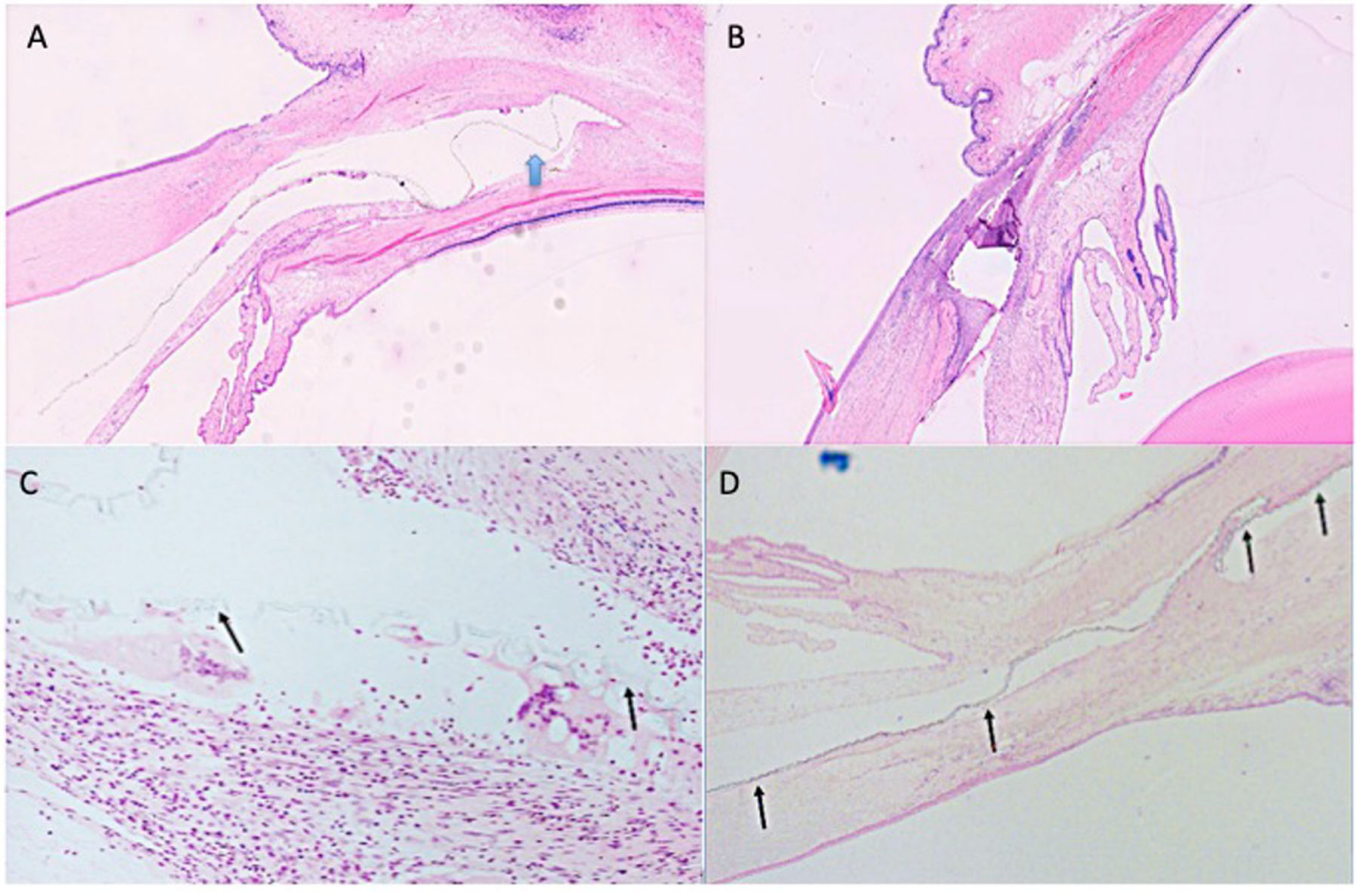
VisiPlate histology. Placement of VisiPlate in the subconjunctival space showing minimal tissue response at 180 days OD (**A**) and OS (**B**). The implant was present, located within a conjunctival pocket and extending into the anterior chamber, close to, but not adhered to, the corneal endothelium. No inflammation or fibrosis was associated with the portion of the implant present in the anterior chamber. Within the pocket, the implant folded once upon itself in one small area. Chronic inflammation (+1), consisting of very small numbers of macrophages and foreign body giant cells, were associated with the portion of the implant present within the pocket, particularly where the implant folded upon itself. Fibrosis and necrosis were not seen. Plastic-embedded sections from vicinity of implant location showing minimal tissue response (**C**). Higher magnification H&E view of implanted eye with giant cells on the surface of the implant (**D**). The implant seen within the anterior pocket at the bottom left of the image and extending into a conjunctival pocket extended up toward the top right with no obvious inflammation around the implant. Arrows show location of VisiPlate

H&E-stained sections taken through the treatment area from untreated controls (93 days) and through the approximate center of the VisiPlate devices (93 and 180 days) were evaluated microscopically (Fig. 5). Three specimens per treatment (three for Group 1 and six for Group 2) and one section per specimen were analyzed. Implants were visible in all treated specimens. For Group 2 eyes that did not receive MMC pretreatment, acute inflammation was observed around all three implants at 93 days, At 180 days, inflammation was lower relative to day 93, with residual inflammatory changes (Fig. 5). The 180-day implants also showed some fibrosis. In contrast, pretreatment with MMC showed no obvious breach of conjunctival tissue and two eyes exhibited minimal chronic inflammation after 180 days, with cell infiltrates composed of macrophages and foreign body giant cells. These cells were associated with the implant surfaces and are not an atypical cellular response to an implant. In the third implant, there was moderate acute inflammation. None of the sections showed fibrosis.

## Discussion

Our study provides data on the efficacy of the novel VisiPlate aqueous shunt device in reducing IOP following implantation into the subconjunctival space of rabbits, and demonstrates safety and biocompatibility of implantation. Current market leaders of IOP lowering devices with long-lasting clinical efficacy have also used this preclinical model and have showed clinical translatability, with fewer rabbits and shorter observation periods [22, 23].

The rabbits tolerated the implantation surgery well and recovered rapidly. VisiPlate’s unique material structure of a continuously corrugated microstructure composite of alumina and parylene-C maintained the mechanical integrity of the device during implantation and in vivo. Typical post-surgical anomalies were exhibited, including ocular irritation, hyphema, conjunctival congestion, swelling, and iritis. These findings were expected post-operative observations, which resolved over the course of the study. The main complication was the development of iris adhesions in eyes implanted with the device, which began around 1 month after device implantation in a subset of eyes. By 3–6 months after device implantation, all eyes had developed such adhesions, which caused distortions of the iris architecture and iris thinning. While in at least one case, an iris injury during device implantation may have contributed to the early development of such adhesions, they were also observed in eyes that had experienced no complications during device implantation. However, these findings were likely due to the unique anatomy of the rabbit eye. This anatomy necessitates localization of the device relatively close to the iris even in cases of proper implantation and placement, which may create a high risk of such adhesions. Therefore, it is unclear to what extent such adhesions would be found in adult humans as the localization of the device farther away from the iris would decrease trauma on insertion, thereby likely decreasing scar tissue formation.

MMC-treated eyes developed enlarged, ischemic blebs. Ischemia and devascularization persisted for ~1 month after device implantation and MMC treatment. After this time, revascularization of the blebs was observed, and bleb size decreased starting 1–2 months after device implantation and MMC treatment. MMC-treated eyes did not differ from eyes not treated with MMC in terms of other ocular anomalies observed. Device implants appeared to function throughout the duration of the study, with confirmed passage of aqueous humor from the anterior chamber to the subconjunctival space via fluorescein testing. Histopathology demonstrated acute inflammation observed around the implants that resolved over time with some fibrosis, expected features of post-implant tissue response. MMC pretreatment may facilitate more advantageous results, as histopathology demonstrated only minimal inflammation on average. Fibrosis resolved by 180 days with some residual acute inflammation in two out of three treated eyes, with no tissue necrosis observed at 93 or 180 days.

IOP decreases in Group 1 device-implanted eyes were relatively minor and not substantially different from a similar decrease observed in untreated eyes during the same time period, apparently due to sporadic variability and drift in IOP over time and poor placement. In contrast, Group 2 animals exhibited a more pronounced decrease in IOP only without MMC, suggesting effectiveness of the device in lowering IOP. MMC-treated eyes initially exhibited slightly greater IOP reductions compared to eyes not treated with MMC, but IOP values in these eyes gradually increased starting in Month 1 and returned to baseline levels by Month 6, while IOP decreases persisted in eyes not treated with MMC. The timeline of this IOP increase suggests that it might be related to the revascularization of the blebs and subsequent bleb failure in the MMC-treated eyes observed around the same time. However, all IOP trends should be interpreted with caution given the small number of eyes examined.

Histopathology data demonstrating minimal inflammatory response confirms the non-fibrotic nature of VisiPlate’s hexagonally-corrugated, parylene-coated surface. Parylene-C has been shown to reduce protein and mice fibroblast adhesion in vitro compared to glass and polystyrene [24], while hexagonal micron-scale surface topography decreases human fibroblast adhesion and proliferation compared to planar surfaces [22]. These features effectively decrease the possibility of clogging and biofouling of the microchannels, as is evident by the IOP decrease of 40–50% and sustained decrease from baseline up to 90 days. Comparatively, XEN and Ahmed glaucoma devices showed decreases in IOP in the 15–40% range, with notes of needing revision surgery to optimize impact [19, 23, 25, 26]. In other literature, glaucoma filtration surgeries tend to fail, and IOP rises, within 2–3 weeks [27, 28], while glaucoma drainage devices fail at 3–6 weeks [29, 30]. It is also important to note the peak IOP found at 120 days. While there is no clear explanation for this observation, it is interesting that a similar peak is present in comparable glaucoma devices such as the XEN and Ahmed. It is possible that a contributing factor may be the pathophysiology of wound healing in rabbit eyes, which are known to aggressively fibrose after surgery [19, 25, 30–33]. Future studies in rabbits will be needed to better understand the underlying pathophysiology of IOP at this distant time point post-surgery. Overall, this preliminary data suggests that VisiPlate’s material and structural properties can improve duration of IOP lowering and reduce otherwise needed revision surgeries.

## Conclusions

The results of this in vivo study demonstrate the biocompatibility and IOP-lowering effect of a novel, multichannel, ultrathin subconjunctival shunt in a New Zealand White rabbit model. The data suggest that VisiPlate may safely enhance aqueous outflow and significantly reduce IOP.

## Supplementary information

Supplementary Table 1

Supplementary Table 2
