# Supplementary material for: Biocompatibility and feasibility of VisiPlate, a novel ultrathin, multichannel glaucoma drainage device
Source: J Mater Sci Mater Med. 2021 Nov 24;32(12):141. doi: 10.1007/s10856-021-06613-8 (PMC8613174; doi:10.1007/s10856-021-06613-8)
Supplement: Supplementary file 1 — Supplementary Table 1 [file 10856_2021_6613_MOESM1_ESM.docx]

**Supplementary Table 1**: Group 1 clinical ophthalmic examinations across categories at time points of post-operative day 0, 1, 3, 7, 28, 51, and 90. According to a modified McDonald-Shadduck scoring system (on a scale in which 0 is categorized as normal), demonstrate great biocompatibility. A) Conjunctival properties (0-3) B) Anterior Chamber Properties (0-4) and C) Iris Properties (0-4) There was no significant changes in corneal involvement, pannus, lens, vitreous flare/cell/hemorrhage, retinal detachment/hemorrhage, or choroidal/retinal inflammation.

| **A) Conjunctival properties** | | | Day 0 (p/o) | 1 | 3 | 7 | 28 | 51 | 90 |
| --- | --- | --- | --- | --- | --- | --- | --- | --- | --- |
| U180 | OD | Discharge | 0 | 0 | 1 | 1 | 0 | 0 | 0 |
|  |  | Congestion | 2 | 2 | 2 | 1 | 1 | 1 | 1 |
|  |  | Swelling | 2 | 2 | 0 | 0 | 1 | 1 | 1 |
|  | OS | Discharge | 0 | 0 | 0 | 0 | 0 | 0 | 0 |
|  |  | Congestion | 0 | 1 | 1 | 0 | 0 | 0 | 0 |
|  |  | Swelling | 0 | 1 | 1 | 0 | 0 | 0 | 0 |
| U189 | OD | Discharge | 0 | 1 | 1 | 0 | 0 | 0 | 0 |
|  |  | Congestion | 2 | 2 | 2 | 1 | 1 | 1 | 1 |
|  |  | Swelling | 2 | 2 | 1 | 0 | 1 | 1 | 0 |
|  | OS | Discharge | 0 | 0 | 0 | 0 | 0 | 0 | 0 |
|  |  | Congestion | 1 | 1 | 0 | 0 | 0 | 0 | 0 |
|  |  | Swelling | 0 | 1 | 0 | 0 | 0 | 0 | 0 |
| U199 | OD | Discharge | 0 | 1 | 1 | 0 | 0 | 0 | 0 |
|  |  | Congestion | 2 | 2 | 1 | 1 | 1 | 1 | 1 |
|  |  | Swelling | 1 | 2 | 1 | 0 | 1 | 1 | 0 |
|  | OS | Discharge | 0 | 0 | 0 | 0 | 0 | 0 | 0 |
|  |  | Congestion | 0 | 0 | 0 | 0 | 0 | 0 | 0 |
|  |  | Swelling | 0 | 0 | 0 | 0 | 0 | 0 | 0 |

| **B) Anterior Chamber properties** | | | Day 0 | 1 | 3 | 7 | 28 | 51 | 90 |
| --- | --- | --- | --- | --- | --- | --- | --- | --- | --- |
| U180 | OD | Aq flare | 4 | 4 | 0 | 0 | 4 | 4 | 0 |
|  |  | Aq cell | 0 | 0.5 | 0 | 0 | 0 | 0 | 0 |
|  | OS | Aq flare | 0 | 0 | 0 | 0 | 0 | 0 | 0 |
|  |  | Aq cell | 0 | 0 | 0 | 0 | 0 | 0 | 0 |
| U189 | OD | Aq flare | 4 | 4 | 0 | 0 | 4 | 4 | 0 |
|  |  | Aq cell | 0 | 0 | 0 | 0 | 0 | 0 | 0 |
|  | OS | Aq flare | 0 | 0 | 0 | 0 | 0 | 0 | 0 |
|  |  | Aq cell | 0 | 0 | 0 | 0 | 0 | 0 | 0 |
| U199 | OD | Aq flare | 4 | 4 | 4 | 0 | 4 | 0 | 0 |
|  |  | Aq cell | 0 | 2 | 0 | 0 | 0 | 0 | 0 |
|  | OS | Aq flare | 0 | 0 | 0 | 0 | 0 | 0 | 0 |
|  |  | Aq cell | 0 | 0 | 0 | 0 | 0 | 0 | 0 |

| **C) Iris involvement** | | Day 0 | 1 | 3 | 7 | 28 | 51 | 90 |
| --- | --- | --- | --- | --- | --- | --- | --- | --- |
| U180 | OD | 1 | 2 | 1 | 0 | 0 | 0 | 0 |
|  | OS | 0 | 0 | 0 | 0 | 0 | 0 | 0 |
| U189 | OD | 1 | 1 | 1 | 0 | 0 | 0 | 0 |
|  | OS | 0 | 0 | 0 | 0 | 0 | 0 | 0 |
| U199 | OD | 0 | 1 | 1 | 0 | 0 | 0 | 0 |
|  | OS | 0 | 0 | 0 | 0 | 0 | 0 | 0 |
