# Supplementary material for: Biocompatibility and feasibility of VisiPlate, a novel ultrathin, multichannel glaucoma drainage device
Source: J Mater Sci Mater Med. 2021 Nov 24;32(12):141. doi: 10.1007/s10856-021-06613-8 (PMC8613174; doi:10.1007/s10856-021-06613-8)
Supplement: Supplementary file 2 — Supplementary Table 2 [file 10856_2021_6613_MOESM2_ESM.docx]

**Supplementary Table 2:** Group 2 clinical ophthalmic examinations across categories at time points of post-operative Day 0, 3, 5, 28, 49, 89, and 180 according to a modified McDonald-Shadduck scoring system (on a scale in which 0 is categorized as normal). A) Conjunctival properties (0-3) B) Anterior Chamber Properties (0-4) and C) Iris Properties (0-4) There was no significant changes in corneal involvement, pannus, lens, vitreous flare/cell/hemorrhage, retinal detachment/hemorrhage, choroidal/retinal inflammation

| **A) Conjunctival properties** | | | Day 0 | 1 | 3 | 5 | 28 | 49 | 89 | 180 |
| --- | --- | --- | --- | --- | --- | --- | --- | --- | --- | --- |
| U546 | OD | Discharge | 0 | 1 | 1 | 0 | 0 | 0 | 0 | 0 |
|  |  | Congestion | 2 | 2 | 2 | 1 | 2 | 2 | 1 | 1 |
|  |  | Swelling | 2 | 3 | 2 | 2 | 1 | 1 | 1 | 1 |
|  | OS | Discharge | 0 | 1 | 1 | 0 | 0 | 0 | 1 | 0 |
|  |  | Congestion | 1 | 2 | 2 | 1 | 1 | 1 | 1 | 2 |
|  |  | Swelling | 1 | 2 | 2 | 1 | 1 | 1 | 1 | 1 |
| U196 | OD | Discharge | 0 | 1 | 0 | 0 | 0 | 0 | 0 | 0 |
|  |  | Congestion | 2 | 2 | 2 | 1 | 1 | 1 | 1 | 1 |
|  |  | Swelling | 2 | 2 | 2 | 2 | 1 | 1 | 1 | 1 |
|  | OS | Discharge | 0 | 1 | 0 | 0 | 0 | 0 | 0 | 0 |
|  |  | Congestion | 2 | 2 | 2 | 1 | 1 | 1 | 1 | 1 |
|  |  | Swelling | 1 | 3 | 1 | 2 | 1 | 1 | 1 | 0 |
| U201 | OD | Discharge | 0 | 1 | 1 | 0 | 0 | 0 | 0 | 0 |
|  |  | Congestion | 1 | 1 | 2 | 1 | 1 | 1 | 1 | 2 |
|  |  | Swelling | 1 | 1 | 2 | 2 | 2 | 1 | 0 | 0 |
|  | OS | Discharge | 0 | 1 | 1 | 0 | 1 | 0 | 0 | 0 |
|  |  | Congestion | 2 | 2 | 2 | 1 | 2 | 1 | 1 | 2 |
|  |  | Swelling | 2 | 2 | 1 | 2 | 2 | 1 | 0 | 0 |

| **B) Anterior Chamber properties** | | | Day 0 | 1 | 3 | 5 | 28 | 49 | 89 | 180 |
| --- | --- | --- | --- | --- | --- | --- | --- | --- | --- | --- |
| U546 | OD | Aq flare | 1 | 1 | 0 | 0 | 1 | 0 | 0 | 0 |
|  |  | Aq cell | 0 | 0 | 0 | 0 | 0 | 0 | 0 | 0 |
|  | OS | Aq flare | 4 | 1 | 1 | 0 | 4 | 0 | 0 | 0 |
|  |  | Aq cell | 0 | 1 | 0 | 0 | 0 | 0 | 0 | 0 |
| U196 | OD | Aq flare | 2 | 2 | 0 | 0 | 4 | 0 | 0 | 0 |
|  |  | Aq cell | 0 | 0 | 0 | 0 | 0 | 0 | 0 | 0 |
|  | OS | Aq flare | 2 | 4 | 0 | 0 | 1 | 0 | 0 | 0 |
|  |  | Aq cell | 0 | 0 | 0 | 0 | 0 | 0 | 0 | 0 |
| U201 | OD | Aq flare | 4 | 4 | 1 | 0 | 0 | 0 | 0 | 0 |
|  |  | Aq cell | 2 | 0 | 0 | 0 | 0 | 0 | 0 | 0 |
|  | OS | Aq flare | 3 | 2 | 0 | 0 | 4 | 0 | 0 | 0 |
|  |  | Aq cell | 0 | 0 | 0 | 0 | 3 | 0 | 0 | 0 |

| **C) Iris involvement** | | Day 0 | 1 | 3 | 5 | 28 | 49 | 89 | 180 |
| --- | --- | --- | --- | --- | --- | --- | --- | --- | --- |
| U546 | OD | 0 | 2 | 0 | 1 | 0 | 0 | 1 | 0 |
|  | OS | 0 | 1 | 0 | 0 | 0 | 0 | 0 | 1 |
| U196 | OD | 1 | 1 | 0 | 1 | 0 | 0 | 0 | 0 |
|  | OS | 0 | 2 | 0 | 1 | 0 | 0 | 0 | 0 |
| U201 | OD | 1 | 1 | 0 | 1 | 0 | 0 | 0 | 0 |
|  | OS | 0 | 0 | 0 | 1 | 0 | 1 | 0 | 0 |
